# Disentangling the co-structure of multilayer interaction networks: degree distribution and module composition in two-layer bipartite networks

**DOI:** 10.1038/s41598-017-15811-w

**Published:** 2017-11-13

**Authors:** Julia Astegiano, Florian Altermatt, François Massol

**Affiliations:** 1 Instituto Multidisciplinario de Biología Vegetal, FCEFyN, Universidad Nacional de Córdoba, CONICET, Argentina; 2 Centre d’Ecologie Fonctionnelle et Evolutive (CEFE), UMR 5175, CNRS - Université de Montpellier - Université Paul Valéry Montpellier - EPHE, 1919 route de Mende, F-34293 Montpellier, France; 3 Eawag, Swiss Federal Institute of Aquatic Science and Technology, Department of Aquatic Ecology, CH-8600, Dübendorf, Switzerland; 4 Department of Evolutionary Biology and Environmental Studies, University of Zurich, CH-8057, Zürich, Switzerland; 5 CNRS, Université de Lille-Sciences et Technologies, UMR 8198 Evo-Eco-Paleo, SPICI group, F-59000 Lille, France

## Abstract

Species establish different interactions (e.g. antagonistic, mutualistic) with multiple species, forming multilayer ecological networks. Disentangling network co-structure in multilayer networks is crucial to predict how biodiversity loss may affect the persistence of multispecies assemblages. Existing methods to analyse multilayer networks often fail to consider network co-structure. We present a new method to evaluate the modular co-structure of multilayer networks through the assessment of species degree co-distribution and network module composition. We focus on modular structure because of its high prevalence among ecological networks. We apply our method to two Lepidoptera-plant networks, one describing caterpillar-plant herbivory interactions and one representing adult Lepidoptera nectaring on flowers, thereby possibly pollinating them. More than 50% of the species established either herbivory or visitation interactions, but not both. These species were over-represented among plants and lepidopterans, and were present in most modules in both networks. Similarity in module composition between networks was high but not different from random expectations. Our method clearly delineates the importance of interpreting multilayer module composition similarity in the light of the constraints imposed by network structure to predict the potential indirect effects of species loss through interconnected modular networks.

## Introduction

Species establish multiple interactions with other species throughout their life cycle. For instance, plants can be attacked by herbivores and seed predators, pollinated by flower visitors and dispersed by birds^1–3^. In some cases, a given organism can also behave as a mutualistic and antagonistic partner of the same species (e.g. adult insects of a given species can behave as pollinators or nectar robbers, or adult insects can act as pollinators while their larvae are herbivores^4,5^). These multiple interactions among species can be integrated in multilayer interaction networks, that is, networks encompassing different types of links between species. Interaction networks often show non-random topological structures and properties^3,6–8^. These properties and structures can affect the ecological and evolutionary dynamics of species assemblages and therefore biodiversity^6,7,9–18^. In this context, one important challenge for network ecologists is to develop tools to analyse the co-structure of multilayer interaction networks^2,19–23^, because such co-structure properties might be key to understand how perturbations (e.g. species loss) can propagate across and between linked networks. Although studies investigating ecological multilayer networks do exist, they all fail to consider similarities of co-structure through a proper statistical framework.

One of the most prevalent patterns found in ecological networks is modular structure. Modular networks emerge when subsets of species interact more among themselves than with other species of the network^24^. Modular structures have been reported in classic food webs^7^, plant-herbivore^10,25^, host-parasitoid^25,26^, plant-pollinator^27,28^, ant-plant^11^, and plant-frugivore networks^29,30^. The modularity of ecological networks may be influenced by features such as interaction type (e.g. antagonistic networks may show higher modularity than mutualistic ones) and intimacy, i.e. the degree of biological integration between interacting individuals (e.g. among plant-ant interactions, non-symbiotic and symbiotic interactions are of low and high intimacy and show low and high modularity, respectively)^10,11,19,31^. Modularity may be more frequently observed in networks of species that establish antagonistic interactions of low intimacy (e.g. herbivory) than in low-intimacy mutualistic networks (e.g. pollination)^10^. However, species-rich mutualistic networks describing low-intimacy interactions may frequently show modular structures (e.g. pollination networks of > 150 species)^27^.

In this article, we present a new comparative method aimed at disentangling the co-structure of multilayer interaction networks, with emphasis on the analysis of network modularity. Since network structure determines the ecological and evolutionary dynamics of multispecies assemblages, understanding the co-structure of interlinked networks is a first key step to unravel the effects that species loss may have on the maintenance of biodiversity^19^. To disentangle the co-structure of multilayer interaction networks, we first propose to compare their distributions of species degree. This degree co-distribution analysis allows understanding the association between the number of interactions (i.e., the degree) that a species establish in one network with the same species’ degree in the other network, and thus helps hypothesize how species loss can propagate through multilayer networks. In this context, we introduce the use of mosaic plots to represent over- and under-representation of species interaction patterns among plants and lepidopterans, advancing the methods proposed in previous studies^2,5,19,22,32,33^. Next, we propose a statistical test to characterize the similarity in module composition between multilayer networks, i.e. in subgroups of species that interact more within groups than among them, using the normalized mutual information of the two classifications of species induced by network modules. The two steps of our method straightforwardly apply to bipartite interaction networks that share species. The most classic example would be organisms that establish different interactions at different life stages, such as herbivorous insect larvae and their pollinating adult stages with plant species. Our comparative analysis can also be extended to study the co-structure of other multilayer ecological networks such as those including pollinators, plants and nectar robbers, and of networks describing spatial or temporal variation of interactions.

We illustrate our method by comparing the co-structure of two Lepidoptera-plant networks from the state of Baden-Württemberg (Germany), one describing low-intimacy antagonistic interactions (i.e. the herbivory network) and the other describing low-intimacy mutualistic interactions (i.e. the flower visitation network). The larvae of most Lepidoptera species (caterpillars) feed on plant tissues, thus establishing antagonistic interactions with plants, whereas the adult lepidopterans visit flowers to feed on nectar and can pollinate them, and thus can act as mutualistic partners^5^. Caterpillars are often characterised by a very particular host plant range and adult lepidopterans often feed on nectar from only a few key flower species^5^. Indeed, sympatric plant species, even if they are closely related, are visited by different moth species^5^. The specificity of antagonistic and mutualistic interactions among plants and Lepidoptera species could be translated into modular structures in interaction networks^34^. Moreover, caterpillars host breadth may influence the number of nectar sources with which adults interact and adults tend to feed on nectar from plant species on which they have fed as larvae^5^. Therefore, similarities in species degree distribution and overlap in module composition among these antagonistic and mutualistic networks can be expected.

## Results

### The co-distribution of species degrees

In both networks, 33% of plant species showed no interaction with Lepidoptera species whereas 7% (herbivory) and 46% (visitation) of Lepidoptera species showed no interaction with plants. Among species showing interactions, most plant and Lepidoptera species interacted with few species (<10 species) whereas few species established interactions with many species (>100 species) in both networks (Fig. S1). Plant species showed similar maximal degree in the herbivory and visitation networks (i.e. interacted with 142 and 161 species, respectively; Fig. S1). Lepidoptera species showed a lower maximum degree at the larval than at the adult stage (i.e. interacted with 70 and 220 species, respectively; Fig. S1).

**Figure 1:**
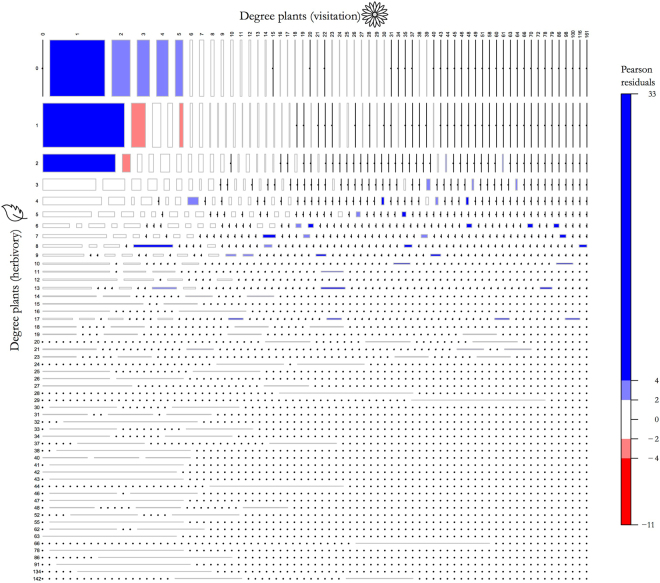
Mosaic plot representing the relative frequency of observed combinations of the number of larval (herbivory) and adult (visitation) Lepidoptera species with which plants interact (i.e. co-distribution of plant degrees). The area of boxes is proportional to the relative frequency of each combination. Boxes are coloured according to the size of Pearson residuals (i.e., the standardized deviations of observed from expected values following Meyer^56^; blue and red boxes indicate combinations of degrees that are over- and under-represented, respectively) and shaded according to the statistical significance of these residuals at approximately α = 0.05 (light blue and light red) and α = 0.0001 (dark blue and dark red). Zero frequency values are represented by a small bullet in order to distinguish them from small frequencies. Designs created by Myly and Lele Saa, for the Noun Project (https://thenounproject.com).

The degree of plant species in the herbivory network was related to their degree in the visitation network (*χ* ^2^ = 7999.7, *df* = 3127, *p* < 2.2e-16; Fig. 1). Species showing no interaction in one network and interacting with few Lepidoptera species in the other network (i.e. with up to five [resp. two] species in the visitation [resp. herbivory] network) were the most frequent and were over-represented among plants (Fig. 1). Plants that interacted with one or two larval Lepidoptera species and one adult Lepidoptera species showed intermediate relative frequency and were under-represented (Fig. 1). Plants interacting with 2 to 17 larval Lepidoptera species and being visited by larger numbers of adult Lepidoptera species were over-represented and showed lower relative frequencies (Fig. 1).

**Figure 2:**
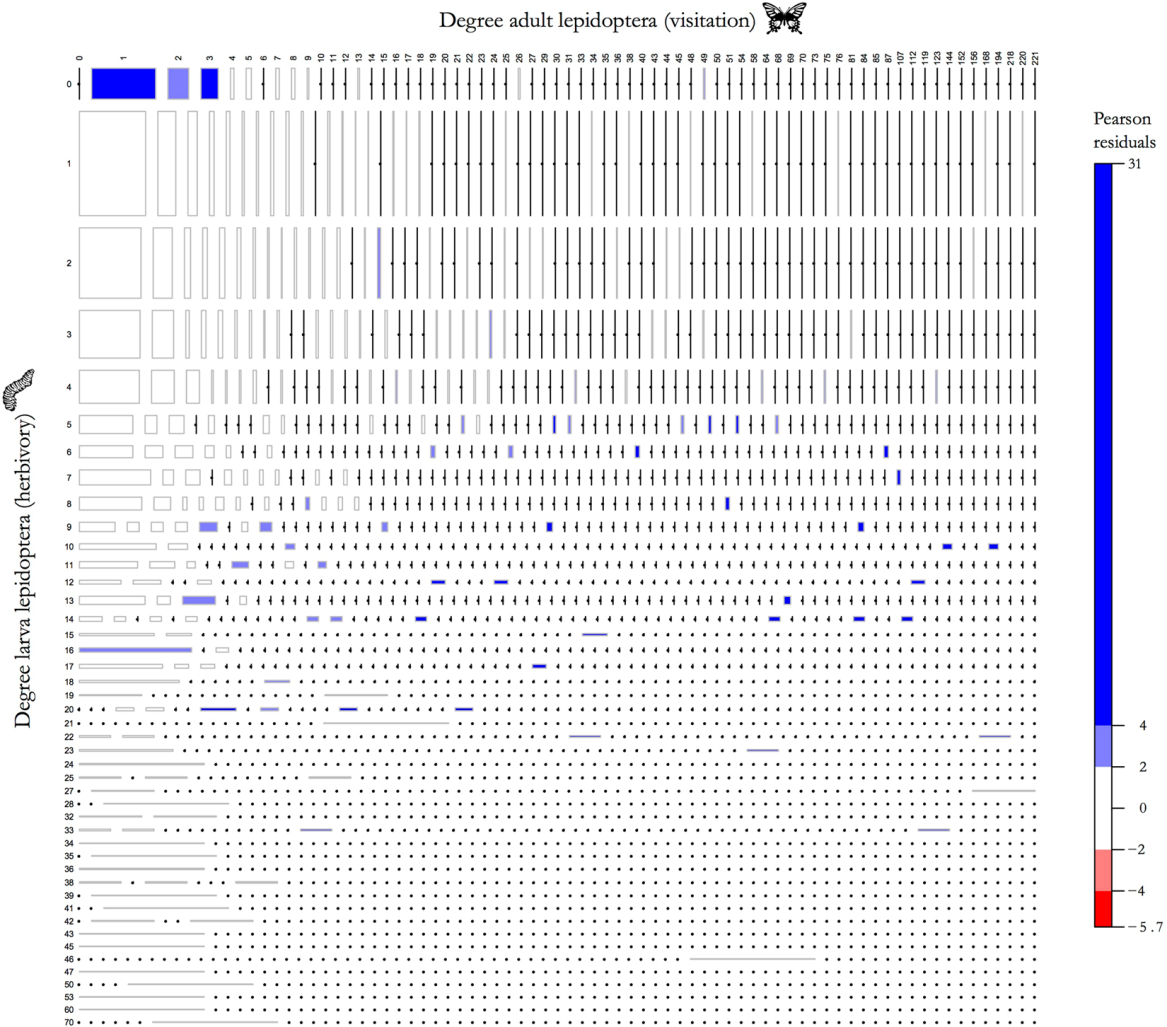
Mosaic plot representing the relative frequency of observed combinations of the number of plant species with which Lepidoptera species interact during their larval (herbivory) and adult (visitation) stage (i.e. co-distribution of Lepidoptera degrees). The area of boxes is proportional to the relative frequency of each combination. Boxes are coloured and shaded according to the size of Pearson residuals and their statistical significance at approximately α = 0.05 (light blue) and α = 0.0001 (dark blue). Blue boxes indicate degree combinations that are over-represented. Zero frequency values are represented by a small bullet in order to distinguish them from small frequencies. Designs created by Cesqo Stefanini and Rachel Siao for the Noun Project (https://thenounproject.com).

The number of plant species with which larval stages of Lepidoptera species interacted was related to the number of plant species visited by their adult stage (*χ* ^2^ = 4571.3, *df* = 2992, *p* < 2.2e-16; Fig. 2). Lepidoptera species showing no interaction with plants in one of the networks and interacting with few plant species in the other network (i.e. with up to three [resp. four] species in the visitation [resp. herbivory] network) were the most frequent and, within this group of Lepidopterans, species only interacting with plants in the visitation network were over-represented (Fig. 2). Species interacting with few plant species at the larval stage and larger numbers of plants at the adult stage (4–7 and 20–123 species, respectively) and those interacting with an intermediate number of plants as larva (8–33 species) and either smaller or larger numbers of species as adults (0–218 species) were also over-represented (Fig. 2). Lepidoptera species interacting with 1 to 4 plants as larva and with one plant as adult were the most frequent among species showing interactions in both stages, but this relatively high frequency was expected (Fig. 2).

### The co-structure of multilayer modular networks: similarity in module composition

**Figure 3:**
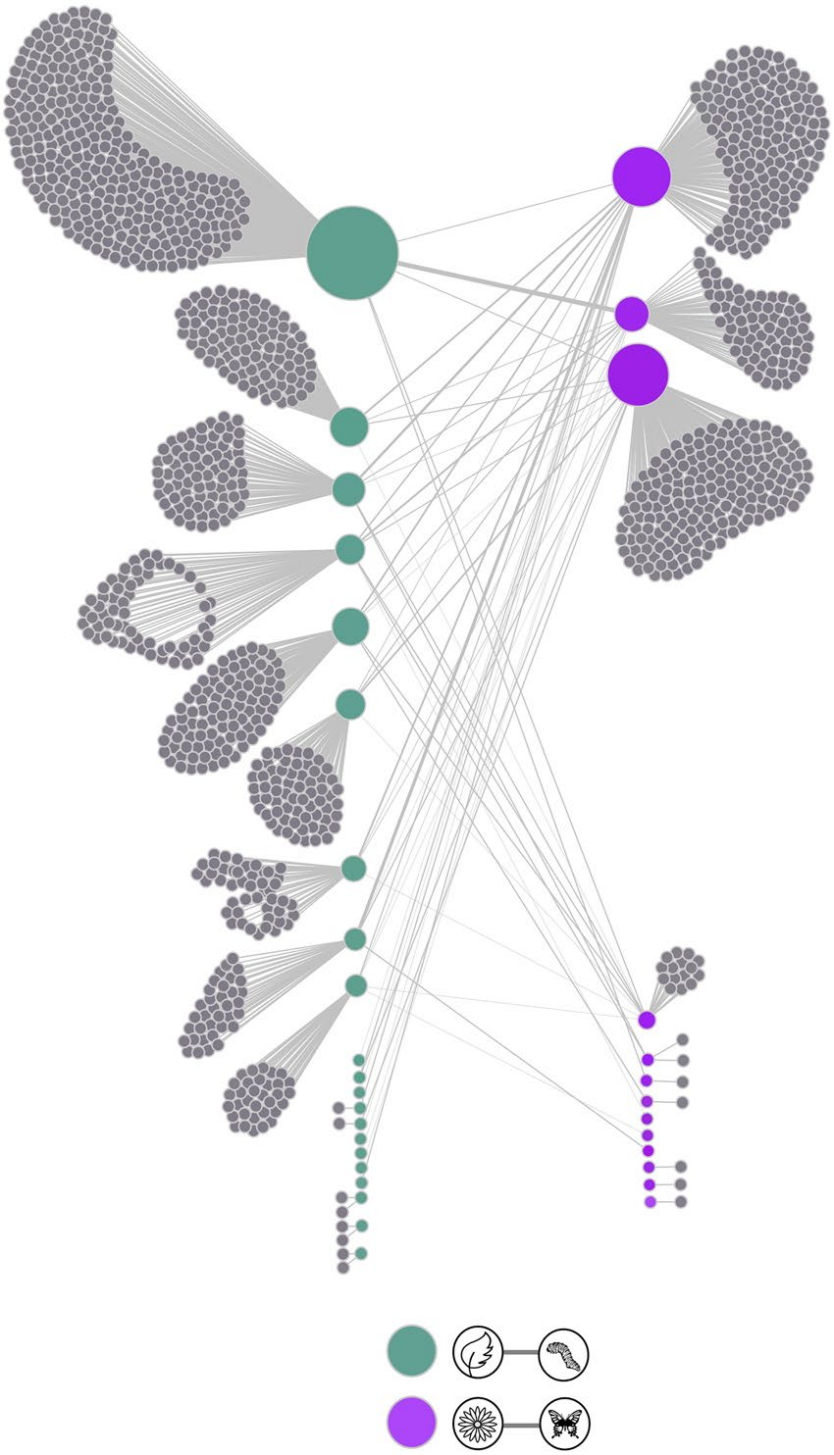
The observed modular co-structure of the herbivory and visitation networks studied. The co-structure is presented as a bipartite network in which nodes represent modules and links denote shared species among modules belonging to the different networks. Link thickness is proportional to the number of shared species. Modules including species that showed interactions are represented as green and purple nodes, respectively. Grey nodes denote modules composed by one species that have no interactions in one of the networks. Modules are vertically ordered by the number of species they comprise. Node size is proportional to the number of modules with which modules interact. Designs created by Myly, LeleSaa, Cesqo Stefanini and Rachel Siao for the Noun Project (https://thenounproject.com).

Species in the herbivory network were grouped in a smaller number of modules (457) than in the visitation network (821). Twenty modules had more than one species in the herbivory network whereas only nine modules had more than one species in the visitation network (Fig. 3). Except for one module of the herbivory network and 4 modules of the visitation network, modules with only one species comprised species that had no interactions (Fig. 3).

Most modules of the herbivory (450) and the visitation network (818) contained species that had no interaction in the other network, i.e. formed one-species modules (range_herbivory_ = 66% to 100% of species per module, range_visitation_ = 31% to 100% of species per module; Fig. 3). Among species showing interactions in both networks, herbivory modules shared species with 1 to 5 visitation modules (11% to 55.5% of visitation modules) whereas visitation modules shared species with 1 to 14 herbivory modules (5% to 70% of herbivory modules; Fig. 3).

The normalized mutual information (*I*) of the classifications induced by modules of the two studied networks was 0.34. This value is not different from the similarity that can be expected between networks with the same number of plant and Lepidoptera species and distribution of the number of interactions per species (range_*Irdm*_ = 0.25 to 0.37; 1st quartile_*Irdm*_ = 0.32, 3rd quartile_*Irdm*_ = 0.35; *p* *=* 0.79).

## Discussion

Disentangling the ecology and evolution of species immersed in multispecies assemblages implies understanding the organization of multiple types of interactions across complex networks^1,15,19^. Thus, developing methods to analyse the structure of multilayer networks should be a priority for the research agenda of network ecologists^2,19–22^. Here we presented a method to evaluate how species interaction patterns change between paired networks by focusing on two key features of network organisation: the degree distribution and the modular structure of ecological networks. The application of our method to Lepidoptera-plant herbivory and visitation networks showed that (1) species that established either herbivory or visitation interactions but not both were prevalent (more than 50% of species) and over-represented among plants and lepidopterans, and were present in most modules in both networks; (2) species whose degree highly increased or decreased from one network to the other were over-represented; (3) similarity in module composition between networks was high but not different from random expectations. Hereafter, we discuss the contributions of our method in relation to existing methodological approaches to multilayer networks and the impact of our results in the light of previous studies on herbivory and visitation networks^2,5,19,22,33,35^. The likely effects of species loss on multispecies assemblage persistence suggested by our results are also discussed to offer new directions for future studies on multilayer networks.

A first step towards understanding how species interact in multispecies assemblages lies in comparing species interaction patterns among ecological networks depicting different interaction types. Most existing studies explored changes in the number of species with which species interact (i.e. their degree) between ecological networks^2,5,19,22,33^. The simplest relationship that can be tested is a correlation between species degrees in these different networks^5,19,22^. Analysing correlations assumes that interactions among organisms may depend on the ability of individuals to detect (mobile organisms) and attract (sessile ones) each other independently of interaction type^19^. For instance, herbivores and nectar robbers may detect plants by recognizing the same phenotypical signals that pollinators do (e.g. floral display), thus plant traits that attract pollinators may also attract herbivores and nectar robbers^19^. Even though correlations may inform about key generalities of species interaction patterns, they may over-simplify them. There are different methods allowing the identification of over- and under-represented interaction patterns. For instance, the distribution of the ratio of degrees in different ecological networks^2,32^ or the rank-degree curves of the same set of species in networks describing different interaction types^33^ can be compared with those obtained in networks constructed under different null models. However, working with degree-ratios may not discriminate between species interacting with few species in both networks (e.g. with 5% of species) from those that interact with several species in both (e.g. with > 90% of species of the network). In the same vein, the visual comparison of rank-degree curves^33^ may be difficult when differences are not so evident, and this procedure lacks a proper statistical test. Moreover, to test if combinations of species degrees are over- or under-represented, randomized networks should maintain the degree distribution of observed networks. Constraining the randomization of observed networks only by maintaining connectance may lead to misleading results since network connectance strongly influences variability in species degree distribution^36^.

Our proposal advances the analysis of interaction patterns in multilayer ecological networks by identifying combinations of species degrees that are over- and under-represented, i.e. that are more or less frequent than expected under the assumption of independent interactions of different types. In the dataset we used for illustration purpose, we found higher prevalence and over-representation of plants with highly asymmetrical combinations of degrees (i.e. those plants which only established interactions with larval or adult lepidopterans and plants that were eaten by few species of herbivores but visited by several species of adult lepidopterans). Extremely specialized plants (i.e. those interacting with few Lepidoptera species in both networks) were under-represented. As far as we know, there are only two datasets exploring the visitation and herbivory interaction patterns of plant species in multispecies assemblages and using the network approach. Pocock *et al*.^3^ studied multispecies interactions in an agroecosystem in England (hereafter the Norwood dataset) and found that some individual plant species were disproportionately well linked to many visitor and herbivore species, but these plants differed between the visitation and the herbivory networks. Sauve *et al*.^22^ explored plant degree correlations in the Norwood dataset and found that the number of flower visitors was positively correlated to the number of herbivores that interacted with plants. Since the reported degree-correlations are low^22^ and different from what can be expected by degree distribution, taken together these results suggest that asymmetrical interaction patterns may be prevalent and over-represented among plants of the Norwood dataset, which is in accordance with our results. Melián *et al*.^2^ studied antagonistic (herbivory) and mutualistic (visitation and frugivory) interactions of the Doñana natural reserve (Spain) and also found that asymmetrical interaction patterns were prevalent among plant species. In the Doñana multispecies assemblage, most plants had low mutualistic-to-antagonistic ratios (i.e. plant species interacted with higher number of herbivores than species of pollinators and seed dispersers) and few had much higher mutualistic-to-antagonistic ratios than expected by chance.

The analysis of the degree co-distribution of larval and adult Lepidoptera species showed that highly asymmetrical foraging interaction patterns were prevalent and over-represented. These patterns included Lepidoptera species that only interacted with plants in the adult stage and those that had specialist larvae and generalist adults or vice versa. Past work comparing the plant as herbivore hosts and nectar sources of species that can be herbivores and floral visitors studied a subset of the data analysed in our study^5^. They found that the number of plant species on which Lepidoptera species feed as larvae was positively correlated with the number of plant species on which adults look for nectar, this relationship being stronger for diurnal Lepidoptera species^5^. Similarly, the degree of adult lepidopterans was also higher for oligophagous and polyphagous species than for monophagous and strictly oligophagous ones in diurnal Lepidoptera species^5^. Our results complement these findings by showing the highly asymmetrical nature of the interactions established by larval (herbivores) and adult (flower visitors) lepidopterans.

As far as data quality is concerned, the plant-insect interaction dataset we used is virtually complete, and thus highly robust (see also Pearse & Altermatt^37^ for an analysis on the robustness of the dataset when removing interactions), but also does not underrepresent the interactions of rare species. Indeed, a correlation between interaction records/degree of interactions and the rarity/commonness of a species would be problematic^38^. However, our dataset is based on the sum of observations of hundreds of entomologists, and rare species often received disproportionate attention (see also^5^), such that plant-insect interactions are very well resolved for all species, regardless of its rarity. In fact, some species may be rare because they have only few interactions, and thus are limited by their host plant use (see Pearse & Altermatt^39^ on that dataset).

Differences between insect herbivores and flower visitors in their level of generalism seem to be widespread. Fontaine *et al*.^40^ studied the interactions established by insect herbivores and flower visitors of species belonging to 44 plant–insect networks describing either visitation or herbivory communities. They found that insect flower visitors tend to interact with far more plant species than herbivores^40^. This difference was mainly attributed to differences in the structure of antagonistic (modular) and mutualistic (nested) networks, which may promote, respectively, the evolution of specialization and generalism^40^ and system stability^10^. We found that adult lepidopterans interacted with higher number of plants than larval lepidopterans. However, by looking at the degree co-distribution of the larval and adult stages of species, we found that higher generalism in herbivores than in flower visitors can also be prevalent and more frequent than expected. Thus, analysing species interaction among modular networks depicting different interaction types may challenge our current understanding of the ecological and evolutionary mechanisms that modulate the distribution of generalism among species^40^.

Understanding the organization of multispecies assemblages also involves analysing similarity of species interaction partners among networks. Similarity analyses at the species level, as performed in previous studies, may be highly informative^2,20,22^. However, as interaction networks show well-defined structures that modulate the ecological and evolutionary dynamics of species interaction patterns^6,7,15^, incorporating network structure into similarity analyses may advance our understanding of the functioning of multispecies assemblages^19,22^. Based on both the high prevalence of modular structures among multispecies assemblages and the idea that modules may be the functional and evolutionary building blocks of ecological networks^7,10,11,27^, we proposed a method to evaluate the similarity in module composition between multilayer networks.

Our analysis involves the characterization of the modular structure of multilayer networks, the assessment of their similarity and the comparison of this similarity with values obtained from randomized networks. Several methods that allow the classification of interacting species in modules (i.e. clustering methods) have been proposed for binary and quantitative networks^41–43^. The ability of the different methods to retrieve module composition may depend on network properties^42^, and there is a limit to the resolution of such methods, i.e. modules under a certain size might be undetectable whatever the method used^44^. The use of the eigenvector-based maximizing modularity algorithm^45^ in our method relies on results of previous studies showing that it is among the clustering methods that best classify species in modules in binary networks, while requiring the lowest computational time^42,45,46^.

There are also several similarity measures to compare the species composition of modules from multilayer networks^46–48^. Measures based on information theory, as the one used in this article, are built on the idea that if species are grouped similarly in two networks, little information is needed to infer the structure of one of the networks given the other^46^. The use of mutual information measures is encouraged because they are not affected by the number and size of modules found in each network as other similarity measures are (e.g. pair counting measures)^48^. As the normalized mutual information index cannot be easily interpreted when it is far from 0 (independent classifications by modules of the two networks) or 1 (same classification by modules of the two networks), network visualization tools may facilitate the analysis of its biological significance, as illustrated by our results.

Network features such as size, connectance and degree distribution can impose constraints on network structure^36,49–51^. Thus similarity in module composition among networks needs to be compared with expectations from random network structure constrained by degree distributions (as performed here), and not with expectations from random network of the same size but with different degree distributions, nor with expectations from random network sharing degree distribution but of different size. Network connectance is negatively correlated with network modularity^10^ and herbivory networks have both lower connectance and higher modularity than visitation networks^10,52^. Thus, it can be expected that among sets of species establishing visitation and herbivory interactions, species within visitation modules may likely be spread among herbivory modules, which may constrain module similarity between networks. Since species degree constrains the interaction pattern of species^53^, the degree distribution of multilayer networks may also limit their similarity in module composition.

Considering the range of similarity values that can be achieved by randomized networks with the same size, connectance and degree distribution, we showed that similarity in module composition between the studied herbivory and visitation networks was high, although not different from random expectations. The importance of degree distribution in predicting the similarity in species interaction partners between herbivory and visitation networks has been little explored in previous studies. In the Norwood dataset, degree distribution predicted that the similarities in flower visitors and in herbivores among pairs of plant species were unrelated^22^. In both the Norwood and the Doñana datasets, the similarity in plant groups between the herbivory and visitation networks was higher than expected by the number of plant groups of each individual network (i.e. groups being subsets of plant species interacting with more similar sets of herbivores and flower visitors)^20^. However, how degree distribution is associated with this similarity remains unknown for the Norwood and Doñana datasets.

Indirect evidence of how widespread the role of degree distribution might be in influencing the similarity of herbivory and visitation networks can be found in the results reported by Fontaine *et al*.^40^. According to this study, herbivore species interacted with plants that were more phylogenetically related than flower visitors did, and plant phylogenetic relatedness was negatively associated with the degree of herbivores, but unrelated to the degree of visitors^40^. In herbivory networks, phylogenetically related plants tend to share modules^25^ and in visitation networks they tend to interact with more similar partners, which also was found for flower visitors^54^. Thus, the difference in the phylogenetic relatedness of plants interacting with herbivores and pollinators and its relationship with species degree^40^ suggests that degree distribution may modulate the structural similarities in species composition between herbivory and visitation networks.

## Future Directions

Disentangling species interaction patterns across multilayer networks may substantially increase our understanding of the effects of species loss on the persistence of multispecies assemblages^5,18,19,22^. Considering the modular structure of networks, it was proposed that high similarity in species composition between modules from networks representing different interactions can lead to the effects of species loss being contained within modules, whereas differences in module composition between networks can help propagate the effects of species loss between networks^19^. Our results on linked herbivory and visitation networks challenge these previous ideas. Most herbivory and visitation modules included species establishing only one interaction type (either herbivory or flower visitation), which may decrease similarity between networks. However, these species represented more than 50% of the assemblage and therefore may likely interact among themselves or with species establishing interactions with few species in both networks (i.e. the second more frequent group). Consequently, the effect of species loss may mostly be contained within modules of a network even when module similarity between networks is low (i.e. low following the ideas proposed by Fontaine *et al*.^19^). Thus, more studies considering the full structure of networks sharing species (i.e. also considering species establishing interactions just in one network) may improve our understanding of the effects of biodiversity loss on multispecies assemblages. In this regard, even when the highest similarity allowed by the constraints imposed by network structure is reached (high similarity following our ideas) interconnecting species with asymmetric interaction patterns may propagate the effects of species loss between networks. The inclusion of interaction strength patterns in those studies may be crucial to predict how interconnecting species forming similar modules between networks can affect the persistence of multispecies assemblages in the face of high biodiversity loss. For instance, stronger dependence among sets of interconnecting species that belong to the same modules in both networks (i.e. those species highly contributing to module similarity) may suggest that their loss is less likely to cause impacts propagating to other modules. Finally, as the studied networks depict interactions across a region, similarity in module composition between networks can reflect interactions among species restricted to certain habitats^5,55^. Therefore, the next steps towards understanding the persistence of multispecies assemblages should focus on the role of habitats in determining the modularity of networks depicting different interaction types.

## Methods

### The co-distribution of species degrees

#### Adjacency matrices and species degree

To evaluate the co-distribution of species degrees in two paired networks sharing species, we first need to construct the matrix defining all the interactions, then obtain the sub-matrices depicting each interaction network and finally calculate the degree of each species in each sub-matrix. In our example of the co-structure of the Lepidoptera-plant herbivory and visitation networks from the state of Baden-Württemberg in south-western Germany, we obtain the matrix defining all the interactions between plants and lepidopterans and then the sub-matrices depicting the herbivory (larvae-plants) and the visitation (adults-plants) networks. Let **A** be the complete adjacency matrix containing all interactions between adult lepidopterans, plants and larvae. In this matrix, 1 or 0 represents the presence or absence of an interaction, respectively. We assume that there are *m* species of Lepidoptera (both at the larval and adult stages) and *p* species of plants. If species are entered in rows and columns and ordered as adult lepidopterans, plants and caterpillars (with the same species order within the two life stages of lepidopterans), the general structure of **A** is:

1 $${\bf{A}}=(\begin{array}{ccc}0 & {\bf{B}} & 0\\ {{\bf{B}}}^{{\bf{T}}} & 0 & {{\bf{C}}}^{{\bf{T}}}\\ 0 & {\bf{C}} & 0\end{array})$$

where **B** is the *m* *×* *p* matrix describing interactions between adult lepidopterans and plants and **C** is the *m* × *p* matrix describing interactions between larvae and plants (i.e. matrices **B** and **C** are the bipartite incidence matrices describing how Lepidoptera and plant species interact), and 0 denotes a matrix of its corresponding shape (*m* × *m* for adult lepidopterans and larvae, *p* × *p* for plants), that is filled with zeros. The (*m* + *p*) × (*m* + *p*) adjacency sub-matrices within the two bipartite networks are:

2 $${\bf{U}}=(\begin{array}{cc}0 & {\bf{B}}\\ {{\bf{B}}}^{{\bf{T}}} & 0\end{array})$$

3 $${\bf{W}}=(\begin{array}{cc}0 & {\bf{C}}\\ {{\bf{C}}}^{{\bf{T}}} & 0\end{array})$$

In sub-matrices **U** and **W**, the first *m* rows and columns refer to lepidopterans (either adults or larvae) while the last *p* rows and columns refer to plants.

Because matrices **U** and **W** are symmetric, the degree *d*_*i*_ of node *i* in matrix **U** (or **W**) can be defined as:

4 $${d}_{i}({\bf{U}})={{\sum }_{j}u}_{ij}={{\sum }_{i}u}_{ji}$$

#### Degree co-distribution

To understand structural similarities between networks depicting interactions established by the same species pool, first we relate *d*_*i*_ (**U**) to *d*_*i*_ (**W**), i.e. the degrees of plants and lepidopterans in both networks. We illustrate this analysis using the degrees of Lepidoptera species, i.e. the values of *d*_*i*_ for 1 ≤ i ≤ *m*. Let the contingency table of degrees be defined as *K*_*u*,*w*_ = |*i*, [*d*_*i*_(**U**) = *u*] & [*d*_*i*_ (**W**) = *w*]|, i.e. *K*_*u*,*w*_ is the number of Lepidoptera species that have degrees *u* and *w* as adults and larvae respectively. We define *p*_*k*_ (**U**) as the empirical probability that an adult lepidopteran has degree *k* (and *p*_*k*_ (**W**) as the matching probability that a larva has degree *k*):

5 $${p}_{k}({\bf{U}})=\frac{{\sum }_{w}{K}_{k,w}}{{\sum }_{u,w}{K}_{u,w}}$$

If degrees from both networks were independent, the probability *p*_*u*,*w*_ that any Lepidoptera species had degrees *u* and *w* as an adult and larva, respectively, would be given by:

6 $${p}_{u,w}={p}_{u}({\bf{U}})Pw({\bf{W}})$$

Based on equation (6), the contingency table *K*_*u*,*w*_ can be compared to its expected proportions through a χ^2^ test or any other similar test of association based on contingency tables. The contribution of each combination of degrees to the overall result of the test can be analysed through its Pearson residuals (the standardized deviations of observations from expected values)^56^. Observed frequencies and Pearson residuals can be represented in a mosaic plot^56^, which can help identify over- and under-represented degree combinations. In a mosaic plot, the frequencies given by a contingency table are portrayed as a collection of rectangular boxes whose areas are proportional to the cell frequencies and can be coloured and shaded to portray Pearson residuals^56^. The construction of mosaic plots for multiple categorical variables is already implemented as the function ‘mosaic’ in the ‘vcd’ package under R^56^.

### Module similarity

Once the co-distribution of species degrees is analysed, we know how species vary in their level of ecological specialization among networks, and whether this variation differs from random expectation. However, this first analysis gives us little information on the structure of these interactions in both networks. The next logical step is thus to look for a simplified description of both networks, i.e. describing a network as a set of vectors, and to assess whether the vectors summarizing the structure of one network explain well the vectors summarizing the structure of the other network. Vectors summarizing information on a given network should, in principle, be as informative as possible on the groups of species that preferentially interact with one another. Based on these vectors, we need to obtain a test statistic that summarizes the level of co-structure between the two networks. Any such statistic can then be tested using the configuration model as the model generating the null hypothesis, i.e. assuming that the expected value for this statistic can be obtained by randomizing species interactions while keeping species degrees equal to their observed values.

#### Module grouping co-structure

One possibility to construct vectors summarizing information on the structure of each network is to obtain a classification of groups of species interacting more among themselves than with species from other groups, i.e. using a module-searching algorithm (e.g. like those described in Newman^45^). This procedure allows obtaining a classification of nodes, i.e. a categorical variable stating that node *i* is to be considered as part of module *g*. The associated disjunctive table, with species as rows and groups as columns, yields 1 if a given species is part of a given group and 0 otherwise. We can then assess the level of similarity between the classifications of species due to the module searches on different networks through the *normalized mutual information*, noted *I* ^47^. This metric assesses whether two classifications on the same set well explain one another. In practice, the *normalized mutual information* is based on the *confusion matrix* **N**, in which rows correspond to the modules found in one of the networks (e.g. the herbivore network of the state of Baden-Württemberg) and the columns correspond to the modules found in the other network (e.g. the visitation network of the same studied region). The element *N*_*ij*_ of the confusion matrix is the number of species that are shared by module *i* of the herbivore network and module *j* of the visitation network, *N*_*i*._ is the sum over all columns *j* of the *N*_*ij*_ elements and represents the size of module *i* of the herbivore network and *N*_.*j*_ is the sum over all rows of the *N*_*ij*_ elements and represents the size of module *j* of the visitation network. *N* is the total number of species. The normalized mutual information measure *I* is calculated as:

7 $$I(H,V)=\frac{-2{\sum }_{i=1}^{{m}_{H}}{\sum }_{j=1}^{{m}_{V}}{N}_{ij}\,\mathrm{log}(\frac{{N}_{ij}N}{{N}_{i}{N}_{j}})}{{\sum }_{i=1}^{{m}_{H}}{N}_{i.}\,\mathrm{log}(\frac{{N}_{i}}{N})+{\sum }_{j=1}^{{m}_{V}}{N}_{j}\,log(\frac{{N}_{j}}{N})}$$

where *m*_*H*_ is the number of modules found in the herbivory network and *m*_*V*_ is the number of modules found in the visitation network. When the two classifications coincide, then *I* (*H*, *V*) takes its maximum value of 1. When the classifications are so different that they are effectively independent (i.e. when *N*_*ij*_ = *N*_*i*._ *N*_*j*_/*N*), then *I* (*H*, *V*) = 0. The calculation of *I* is already implemented through function ‘compare.communities’, option = nmi, in the ‘igraph’ package under R.

The natural ‘null model’ to test for the observed value of *I* is the configuration model, i.e. randomizing both networks through permutations of interactions while keeping degrees equal to their observed values. By constructing such random bipartite networks, we generate a distribution of values for *I* under this null model. Comparing the observed value of *I* to this null distribution will tell us whether and how the congruence of the two classifications induced by the modules of the two bipartite networks differs from what can be expected from random bipartite networks with the same distribution of species degrees. The R version of the code to analyse module similarity between networks following the method proposed in this section is available as supplementary information (Appendix 1).

#### Construction of random bipartite networks constrained by degree distribution

To generate random bipartite networks constrained by degree distribution, we use the *curveball* algorithm recently developed by Strona *et al*.^57^ (see Appendix 1, Supplementary information). This algorithm randomly selects pairs of species within a given trophic level, looks for exclusive interacting partners of each species and exchanges them between these species. After this procedure has been repeated many times, the randomized set of species per interacting partner lists are used to recompile the new presence-absence matrix.

### Application: the co**-**structure of plant-lepidopteran networks

*Data set*. In this study, we used two large bipartite networks of interactions between Lepidoptera and plant species from the state of Baden-Württemberg (Germany). The dataset has been compiled and studied by F. Altermatt and I. Pearse^5,37,39,58^. Lepidoptera species represented >90% of all resident Macrolepidoptera species that have ever been recorded in the Baden-Württemberg state^55^. Information on plant species with which Lepidoptera species interact was obtained from published work^55^. We considered all terrestrial vascular plant species found in Baden-Württemberg as potential hosts, based on the published plant database http://www.floraweb.de/ ^58^.

The dataset used herein is based on an extensive compilation of semi-quantitative estimates of plant-insect interactions, which were assembled by hundreds of entomologists in Baden-Württemberg (for details see Ebert^59^). The data set is based on in total of 2,149 million adult individuals (nectaring visits) and 2,342 million larval individuals (herbivory records) recorded for all these Lepidoptera species (excerpt of the database of Ebert^59,60^; see also Altermatt & Pearse^5^). Thereby, it may be one of the most complete plant-insect interaction datasets. Information on the magnitude/abundance of an interaction is on an ordinal scale, and the dataset is especially complete for rare species, given that those have often received disproportionate attention by entomologists. Thus, rarity of a species does not drive a species degree distribution, but the degree distribution is especially well resolved for rare species (for details see also^5,37,39,58^). Furthermore, the use of host plants is especially well resolved for specialist species, while the interactions for the trophically most generalist species are not completely covered. The dataset is highly robust to rarefaction/subsampling. For example, Pearse & Altermatt^37^ showed that the removal of up to 80% of the observed interactions does only marginally reduce the predictability of novel host plants uses.

The herbivory network depicts interactions between caterpillars and the plant species they feed on (antagonistic interactions). The visitation network depicts Lepidoptera species visiting plants to feed on nectar and potentially pollinate them (mutualistic interactions). Since our objective is to describe similarity among modules of the herbivory and visitation networks, we include all Lepidoptera and plant species in the two networks (e.g. Lepidoptera species interacting with some plants in the herbivory network but with no plant in the visitation network were included as species with zero degree in the visitation network). Among species with no interaction in the visitation network were some lepidopterans that do not have a functional proboscis, and thus only interact as herbivores with host plants. A lack of a larval interaction is due to missing observations, or (for a few cases) interactions with non-vascular plants only as larval host plants.

Interaction networks describe 11,533 interactions among 972 Lepidoptera species (from 25 families) and 1,123 plant species (from 103 families). Of those, 5,219 comprise interactions of caterpillars and their host plants and 6,314 comprise interactions with adult lepidopterans and nectar source plants. Network connectance was calculated as $C=\frac{L}{AP}$, with *L* being the number of interactions and *A* and *P* the number of animal (Lepidoptera) and plant species, respectively, and was higher in the visitation than in the herbivory network (*C*_*V*_ = 0.006, *C*_*H*_ = 0.005).

The modular structure of the herbivory and the visitation networks was estimated by maximizing modularity i.e. partitions of the original graph were allowed only if they increased modularity^45^. The modular structure after each partition was characterized by calculating the leading non-negative eigenvector of the modularity matrix of the graph^45^. We used the package ‘igraph’ under R. After identifying module composition in both networks, we obtained the normalized mutual information (*I*) as described above (see Appendix S1, Supplementary information).

### Data availability statement

R scripts and data are available as online supplementary information.

## Electronic supplementary material

Supplementary information

Dataset 1

Dataset 2
