# Supplementary material for: Disentangling the co-structure of multilayer interaction networks: degree distribution and module composition in two-layer bipartite networks
Source: Sci Rep. 2017 Nov 13;7:15465. doi: 10.1038/s41598-017-15811-w (PMC5684352; doi:10.1038/s41598-017-15811-w)
Supplement: Supplementary file 1 — Supplementary information [file 41598_2017_15811_MOESM1_ESM.pdf]

Supplementary information for

Disentangling the co-structure of multilayer interaction networks: degree distribution and module composition in two-layer bipartite networks

Julia Astegiano*, Florian Altermatt and François Massol

*Corresponding author: Julia Astegiano (juastegiano@gmail.com)

Appendix S1

Module similarity. The following R-script allows the analysis of module composition similarity in multilayer networks as described in the main text.

```
rm(list=ls( ))

library(igraph)
library(FactoMineR)
library(scatterplot3d)
library(permute)
library(lattice)
library(vegan)

#Loading herbivory and visitation networks
herbiv<-data.matrix(read.table(file.choose(),sep=";",dec=",",header=FALSE))
visit<-data.matrix(read.table(file.choose(),sep=";",dec=",",header=FALSE))
x=list(herbiv, visit)

#function to randomize the observed networks
aleatoriz<-function(matrix1, matrix2) {
	list(nullmodel(matrix1,"curveball"),nullmodel(matrix2,"curveball"))
	}

#Function to obtain the statistic I, i.e. the normalized mutual information (Danon et al. 2005)
normutinfo<-function(matriz) {
	m1<-matriz[[1]]
	m2<-matriz[[2]]
incidenceherbiv<-graph.incidence(m1)
adjacencyherbiv<-get.adjacency(incidenceherbiv)
edgelistadjherb<-graph.adjacency(adjacencyherbiv)
igraph.arpack.default$maxiter=100000000
```

```
modherbiv<-cluster_leading_eigen(edgelistadjherb, options = igraph.arpack.default)
vectormodherbv<-modherbiv$membership
incidencevisit<-graph.incidence(m2)
adjacencyvisit<-get.adjacency(incidencevisit)
edgelistadjvisit<-graph.adjacency(adjacencyvisit)
modvisit<-cluster_leading_eigen(edgelistadjvisit, options = igraph.arpack.default)
vectormodvisit<-modvisit$membership
idanon<-compare(comm1=vectormodherbv, comm2=vectormodvisit, method="nmi")
}

#Obtaining the value of I for the observed networks
idanonobs<-normutinfo(x)

#Function to obtain randomized herbivory and visitation networks and the I value of
multilayer networks
idanonrandom<-function(xx, method = aleatoriz, estad = normutinfo, nsimul = 9999) {
        irandom<-matrix(0, 1, nsimul)
                for (i in 1:nsimul) {
                irandom[1, i]<-estad(method(xx[[1]], xx[[2]]))
                }
                return(irandom)
                irandom
         }

#Obtaining the values of I for the randomized networks
idanonranddistrib<-idanonrandom(x)

#statistics
#quantiles
idanonquantiles<-quantile(idanonranddistrib, probs = c(0.025,0.05,0.5,0.95, 0.975))
write.table(idanonquantiles,"quantiles.txt",sep="\t",row.names=F)

tablefr<-data.matrix(idanonranddistrib)

#one-tailed p value
mponethigher<-which(tablefr>=idanonobs)
ponethigher<-(length(mponet)+1)/10000
mponetlower<-which(tablefr<=idanonobs)
ponetlower<-(length(mponet)+1)/10000

#two-tailed p value
m1twot<-abs(tablefr-mean(tablefr))
```

```
m2twot<-which(m1twot>=abs(mean(tablefr)-idanonobs))
ptwot<-(length(m2twot)+1)/10000

results<-data.frame("p.values" = c(ponetlower,ponethigher,ptwot))

write.table(results,"p.values.txt",sep="\t",row.names=F)
```

The co-distribution of species degrees

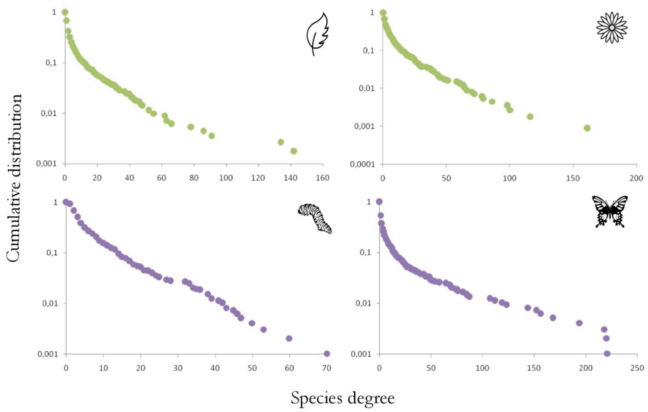

Figure S1. Linear-log plots of the cumulative distributions of species degrees for plant and Lepidoptera species in the herbivory and visitation networks. Designs created by Myly, LeleSaa, Cesqo Stefanini and Rachel Siao for the Noun Project (https://thenounproject.com).
